# Impact of Heat–Moisture Treatment on Multi-Scale Structure, Functional Properties, and In Vitro Digestion of Low-Glycemic-Index Rice Starch

**DOI:** 10.3390/foods15111960

**Published:** 2026-06-02

**Authors:** Rong Zhou, Jingyi Zheng, Zhengyu Jin, Tao Zhang, Ming Miao

**Affiliations:** State Key Laboratory of Food Science and Resources, Jiangnan University, Wuxi 214122, China; 6230112123@stu.jiangnan.edu.cn (R.Z.); jingyizheng218@163.com (J.Z.); fpcenter@jiangnan.edu.cn (Z.J.); zhangtao@jiangnan.edu.cn (T.Z.)

**Keywords:** heat–moisture treatment, rice starch, multi-scale structure, functional properties, in vitro digestibility

## Abstract

This study examined the effect of heat–moisture treatment (HMT) at 100, 110, or 120 °C for 10 min on the multi-scale structure, functional properties, and in vitro digestibility of the starch isolated from low-GI rice. The HMT decreased the leaching rate and swelling power of the starch, compared to those of the untreated starch. The relative crystallinity decreased, and the short-range molecular order increased as the treatment temperature increased from 24.3% to 15.8%. The weight-average molecular weight decreased from 1.7 × 10^7^ g/mol to 0.9 × 10^7^ g/mol with a broad molecular weight distribution and increased particle size. The proportion of the short amylopectin chains (DP 6–12) increased while that of the long chains (DP > 36) decreased. Mild HMT (100 °C) increased gel strength, while higher-intensity HMT (110–120 °C) reduced G′ as measured by rheology. The content of rapidly digestible starch in rice starch was reduced from 68.7% to 57.4%, slowly digestible starch was increased from 24.5% to 26.8%, resistant starch was enhanced from 6.9% to 15.8%, and the digestion lag time was prolonged to 28.62 min. The in vitro digestion assays indicated that the estimated glycemic index of starch treated at 120 °C was 48.80. This sample exhibited the highest suppression of digestibility. It also showed the highest increase in resistant starch content. The results provide a theoretical ground for low-GI product development from HMT modified rice starch.

## 1. Introduction

White rice serves as the primary food source for over 50% of the global population. During the prolonged milling process, the starchy endosperm is fully exposed and could be easily gelatinized and rapidly digested. Such rice typically provides a high postprandial glycemic response. Consistently consuming this variety of rice over a long period was significantly linked to a heightened risk of developing type 2 diabetes [1,2]. Physical modification to decrease the digestion rate and glycemic effect of rice starch is an active research topic in food science and nutritional health. Heat–moisture treatment (HMT) is an eco-friendly physical approach that does not involve the use of chemical additives. During HMT, starch undergoes several structural changes. Amylopectin branches are partially split, increasing the fraction of short chains (DP 6–12) and decreasing the weight-average molecular weight of amylopectin [3,4]. While HMT usually reduces the relative crystallinity, it has been reported that HMT-treated starch had increased absorbance between the 1047 cm^−1^ and 1022 cm^−1^ bands. This phenomenon was interpreted to indicate that, if long-range crystalline order was disrupted, then the short-range helical structures became strengthened [5,6]. HMT could also result in the formation of surface indentations on starch granules and lead to inter-granular aggregation and surface compaction [7,8].

To a great extent, how much HMT could restructure the starch digestibility depended on HMT’s ability to induce structural changes of the starch, occurring on different scales. The multi-scale architecture of starch is inherently linked to its physicochemical properties: gelatinization, retrogradation, and gelation. These properties would ultimately determine how much of the starch is made available to the digestive enzymes. Under conditions of extremely low humidity and elevated temperatures, HMT utilizes the combined effects of heat and moisture to enhance the organization of the starch molecular chains. Such a rearrangement would slow down enzymatic hydrolysis and increase the yield of resistant starch [9,10]. Packaged tightly, double helices could limit the irreversible swelling of granules and reduce the leaching of amylose during heating. As a consequence, gel network formation upon cooling was minimized in strength and extent, and retrogradation was inhibited [11]. A granular surface with increased tightness might also function as a physical barrier. The inward diffusion of amylolytic enzymes and their subsequent binding to the starch substrate were impeded, resulting in a slowed overall rate of enzymatic digestion [12]. It is therefore essential to establish quantitative connections between HMT-driven structural modulation and digestive function in order to rationally design low-GI starch products. Most of the existing work, however, has investigated single treatment conditions. Much remains to be done in clarifying the regulatory mechanisms that act under gradient HMT, as well as the intrinsic connections between structural alteration and final functional performance.

The current work includes an extensive and systematic characterization of the starch isolated and purified from HMT-treated rice. Various structural features were assessed: molecular weight and chain length distribution, crystalline and short-range ordered structure, and particle size distribution. The gelatinization properties, the thermodynamic properties, and the gel rheological behavior of the starch were determined. The in vitro digestion kinetics of native and fully gelatinized samples were studied, and the distribution of nutritional fractions was determined. The objective was to find quantitative relationships between the HMT processing conditions and the multi-scale structural as well as functional properties of starch. The results could serve as theoretical guidance for the design of HMT-treated starch products with a low glycemic index.

## 2. Materials and Methods

### 2.1. Materials and Reagents

Low-GI rice was purchased from Nanjing Jirui Hongbang Trading Co., Ltd. (Nanjing, China). Its initial moisture content was 12.46%, starch content was 74.92%, protein content was 8.83%, and amylose content was 25.6%. The measured glycemic index of this rice was 43.59, which classifies it as low GI (GI ≤ 55) according to the international standard (ISO 26642:2010). After HMT, all samples were air-dried to a final moisture content of 14% before further analysis. To determine the moisture content of the sample by oven drying, the temperature set to 105 °C and dried until a constant weight was reached (approximately 6 h). The rice was stored in darkness at temperatures below 4 °C. Storage duration did not exceed three months. This protocol prevented moisture uptake, odor contamination, and pest infestation. Uniformity and stability of physicochemical properties were thereby ensured. Porcine pancreatic α-amylase (No. E-PANAA-3G, 75,000 U/g) was acquired from Megazyme International Ireland (Wicklow, Ireland). Amyloglucosidase derived from Aspergillus niger (A7095, ≥260 U/mL, aqueous solution) was obtained from Sigma-Aldrich Chemical Co. (St. Louis, MO, USA). All other chemical reagents were of analytical grade and were procured from Sinopharm Chemical Reagent Co., Ltd. (Shanghai, China).

### 2.2. Starch Extraction

Rice starch was extracted according to the procedure of Li et al. [13] with minor modifications. First, 10 g of starch was treated with 50 mL of 80% by volume aqueous ethanol and stirred at ambient temperature for 30 min to eliminate soluble sugars and unbound lipids. The mixture was subsequently centrifuged at 3000× *g* for 10 min. The supernatant was removed and the pellet was resuspended in 50 mL of a 0.2% (*w*/*v*) sodium hydroxide solution, then stirred at 4 °C for 12 h to extract any remaining proteins. The suspension was neutralized with 0.1 mol/L hydrochloric acid and centrifuged with the same settings. The resulting starch pellet was washed successively with deionized water, 70% (*v*/*v*) ethanol, and absolute ethanol, dried at 40 °C for 24 h, and stored in a desiccator until needed.

### 2.3. Heat–Moisture Treatment

The initial moisture content of the rice starch was determined by oven-drying at 105 °C to constant weight. The starch was then adjusted to 25% moisture by adding the calculated amount of distilled water as a fine spray with continuous mixing. The moistened starch was mixed, sealed, and equilibrated at 4 °C for 24 h. Thus, all samples reached the same final moisture content (25%) before autoclaving (Model YXQ-LS-50SII, Boxun Industrial Co., Ltd., Shanghai, China). Then, 10.0 g of starch was transferred into sealed pressure-resistant glass tubes (30 mL, screw caps with silicone gaskets). The tubes were placed in a vertical autoclave and heated at 100, 110, or 120 °C for 1 h, yielding samples designated HMT-1, HMT-2, and HMT-3, respectively. After heating, the autoclave was cooled naturally to below 40 °C. The tubes were then opened after reaching ambient temperature, and the samples were dried at 40 °C for 24 h. The untreated raw starch was used as a control (HMT-0). Finally, the dried samples were ground and passed through a 200-mesh sieve for further use.

Amylose leaching was quantified by following Yin et al.’s [14] method. A 2% (*w/w*) dispersion of the sample was prepared, and its temperature was raised to 95 °C in a water bath and held for 10 min, with occasional shaking. Once cooled to ambient temperature, the mixture underwent centrifugation at 5000× *g* for a duration of 10 min. The supernatant was subsequently collected and blended with 6 mL of 0.33 mol/L NaOH, then heated once more at 95 °C for 30 min. A 0.1 mL sample was taken, and the pH was modified to around 5.5 with 1 mol/L HCl. The sample volume was adjusted to a final volume of 100 mL using distilled water. Following the addition of 0.05 mL of 0.01 mol/L I_2_-KI reagent, the sample was allowed to sit at ambient temperature for 30 min, after which the absorbance was recorded at 620 nm. A standard curve was created using potato amylose standards, and the amylose elution ratio was derived from this standard curve. Following that, leaching rate (W_1_) and swelling rate (W_2_) were calculated as per the following equations (both reported as the ratio of precipitate mass (after centrifugation) to the initial sample mass):

(1) $$W_{1}/\%\;=\;\frac{C\;\times \;100}{m}\;\times \;100$$

(2) $$W_{2}/\%\;=\;\frac{m_{2}}{m_{1}\;\times \;(1\;-\;W_{1}/100)}\;\times \;100$$

where C is the mass of amylose (mg) determined from the standard curve; m is the dry weight of the sample (mg); m_1_ is the dry weight of the sample (g); and m_2_ is the mass of the precipitate after centrifugation (g).

### 2.4. Structural Characterizations

Particle size distribution of starch was measured with a laser diffraction particle size analyzer, following the method of Bohačenko et al. [15] with slight modifications. A 100 mg portion of starch was weighed and dispersed in 10 mL of deionized water. After thorough mixing, the suspension was transferred to the sample cell. The refractive index was set at 1.53 for starch and 1.33 for water.

XRD analysis of the samples was performed according to the method of Dang et al. [16] with slight adjustments. The measurements were taken at 40 kV and 40 mA, utilizing Cu-Kα radiation (λ = 1.54 Å). Diffractograms were obtained over a 2θ range of 5° to 50°. The scanning rate was 4°/min. Before analysis, specimens were conditioned within a controlled temperature–humidity chamber (LHS-80HC, Bluepard, Shanghai, China). Conditioning was continued until a moisture level of 12% was attained. Relative crystallinity was determined utilizing MDI Jade version 6.5 software (Materials Data Inc., Livermore, CA, USA).

FT-IR spectra were recorded on a Nicolet iS10 spectrometer (Thermo Fisher Scientific, Waltham, MA, USA) equipped with an attenuated total reflectance accessory. Spectra were collected over the wavenumber range of 4000 cm^−1^ to 500 cm^−1^ at a resolution of 4 cm^−1^, with 32 scans accumulated per sample. A background spectrum taken against air was acquired before each sample run, as described in the literature [17].

The distribution of chain lengths in amylopectin was analyzed using high-performance anion-exchange chromatography in conjunction with pulsed amperometric detection, based on the method established by Moreno-Zaragoza et al. [18], with slight modifications. Following the removal of fats and proteins, the sample was treated with heat-stable α-amylase in a boiling water bath for 30 min to selectively hydrolyze amylose [18]. Isoamylase was then added at an activity of ≥500 U/mg, and the mixture was held in a 40 °C water bath for 24 h. This step cleaved the α-1,6-glycosidic bonds of amylopectin, releasing linear oligosaccharides of various chain lengths. The enzymatic hydrolysate underwent inactivation by being gently warmed within a quickly boiling water bath for a duration of 10 min. Afterward, it was permitted to cool down and then went through a membrane filtration unit with a pore size of 0.22 μm.

Separation was carried out on a CarboPac PA100 analytical column (4 mm × 250 mm) using a mobile phase of 150 mmol/L NaOH at a flow rate of 1.0 mL/min. The column’s temperature was maintained at 30 °C. A pulse amperometric detector was used to detect signals of single oligosaccharide components, and degrees of polymerization were assigned by comparing retention time to those of standards of known values. Relative percentages were determined for four chain length ranges by peak area normalization: DP 6–12, DP 13–24, DP 25–36, and DP > 36.

The M_w_ was determined using gel permeation chromatography (GPC) [19]. Starch (10 mg) was dissolved in 10 mL of 0.1 mol/L NaCl solution in a sealed vial and heated at 60 °C in a water bath with magnetic stirring (300 rpm) for 2 h to ensure complete dispersion. The solution was then centrifuged at 10,000× *g* for 10 min, and the supernatant was filtered through a 0.22 µm membrane filter. The filtrate (1 mg/mL starch) was injected into a Sepharose CL-6B column (1.6 × 70 cm, GE Healthcare, Chicago, IL, USA) equilibrated with 0.1 mol/L NaCl at 30 °C. The column was eluted with 0.1 mol/L NaCl at a flow rate of 1.0 mL/min. Eluted components were detected using a differential refractive index detector (RID-20A, Shimadzu, Kyoto, Japan) The column was calibrated with dextran standards (molecular weights: 1 × 10^4^, 5 × 10^4^, 1 × 10^5^, 5 × 10^5^, 1 × 10^6^, 2 × 10^6^ g/mol; Sigma-Aldrich, St. Louis, MO, USA). The M_w_ of each sample was calculated from the calibration curve using Agilent GPC/SEC software (version 1.2, Agilent Technologies, Santa Clara, CA, USA). The reported M_w_ values were relative to the dextran standards.

### 2.5. Functional Properties

Pasting properties of starch were measured in a rapid visco analyzer (RVA Starch Master 2, PerkinElmer, Waltham, MA, USA). The procedure of Xiong et al. [20] was used. Starch (2.5 g, moisture content corrected to 14%) was charged into an RVA canister. The starch was mixed with 25 mL of deionized water. The heating and cooling session lasted for 13 min. The following sequence of steps was used. An initial equilibration at 50 °C was followed by a holding period of 1 min. The temperature was increased to 95 °C at a rate of 12 °C/min. A holding time of 2.5 min at 95 °C was also employed. Cooling down to 50 °C was done at 12 °C/min. A duration of 2 min at a temperature of 50 °C was the last stage of the program to complete the process. For stirring speed, we employed 960 rpm for the first 10 s. This allowed an even dispersion to be achieved. The speed was decreased to 160 rpm for the sedimentation. Pasting parameters registered peak viscosity (PV), trough viscosity (TV), final viscosity (FV), setback viscosity (SV), breakdown viscosity (BV), and gelatinization temperature (GT).

A differential scanning calorimetry instrument (DSC 3 from Mettler Toledo in Greifensee, Switzerland) was utilized to ascertain the thermodynamic properties of the starch. The method of Zhang et al. [21] served as the foundation for this study, although some essential alterations were incorporated. A 3.0 mg mass of fine-grained starch was precisely dispensed into diminutive aluminum DSC pans, each holding a volume of 40 μL. Subsequently, 9 μL of sterile distilled water was gently introduced into every pan. Pans were sealed. Equilibration was allowed to proceed overnight at 25 °C. This was sufficient to ensure that the starch was fully hydrated. An empty sealed reference aluminum pan, devoid of any substance, was designated to counterbalance any potential baseline influences. This DSC examination aimed to illuminate the thermal characteristics inherent to the starch. Samples were subjected to heating from 30 °C to 110 °C, utilizing a heating rate of 10 °C/min. The onset (T_o_), peak (T_p_), and conclusion (T_c_) temperatures of gelatinization were acquired, and the gelatinization enthalpy (ΔH) was calculated by instrument software.

Rheological measurements were performed on an AR-G2 rheometer (TA Instruments, New Castle, DE, USA) equipped with a 40 mm parallel plate geometry. Starch preparation involved an aqueous suspension of 15% purified starch (*w*/*v*) heated in a boiling water bath for 20 min for complete gelatinization. After cooling to 25 °C, the resulting paste was loaded onto the rheometer stage. Frequency sweeps were performed at 25 °C in the linear viscoelastic regime with the strain fixed at 1% over a frequency range of 0.1 to 10 Hz. The reported G′, G″, and tan δ values were taken at a single frequency of 1 Hz (within the linear region). Storage modulus (G′), loss modulus (G″), and loss tangent (tan δ = G″/G′) were recorded.

### 2.6. In Vitro Digestion

The in vitro digestion procedure was conducted according to Li et al. [22], with several important modifications. A finely ground powder (100 mg) was dissolved in 10 mL of sodium acetate buffer (pH 5.2). Following a pre-incubation period at 37 °C for 10 min, digestion commenced with the addition of 10 mL of enzyme mixture containing 290 U of porcine pancreatic α-amylase and 50 U of amyloglucosidase. At time points of 0, 5, 10, 15, 20, 30, 45, 60, 90, 120, and 180 min, 0.05 mL samples were withdrawn and combined with nine volumes of absolute ethanol to halt the reaction. The samples were then centrifuged (8000× *g* for 5 min), and the supernatant’s reducing sugars were assessed using the DNS method: 0.1 mL of the supernatant was mixed with 0.2 mL of DNS reagent, boiled for 5 min, cooled, then combined with 0.9 mL of water and measured at 540 nm. Glucose concentrations were established using a standard curve and digested starch was quantified as glucose multiplied by 0.9, the conversion factor from glucose to starch. Digestion curves were generated, with the amounts of rapidly digestible starch (RDS), slowly digestible starch (SDS), and resistant starch (RS) computed accordingly.

(3) $$\mathrm{RDS}\;(\%)\;=\;{(G}_{20}\;-\;\mathrm{FG})\;\times \;0.9/\mathrm{TG}\;\times \;100\%$$

(4) $$\mathrm{SDS}\;(\%)\;=\;{(G}_{120}\;-\;G_{20})\;\times \;0.9/\mathrm{TG}\;\times \;100\%\;$$

(5) RS (%) = (1 − RDS − SDS) × 100%

where $G_{20}$ and $G_{120}$ represent the quantity of glucose produced (mg) at 20 and 120 min of hydrolysis, respectively. FG indicates the free glucose level in the sample (mg), while TG denotes the total starch weight on a dry weight basis (mg).

The HI and eGI of rice were calculated following the method described by Li et al. [23], and the eGI was calculated using the equation proposed by Goñi et al. [24] with white bread as a reference:

(6) $$\mathrm{HI}\;(\%)\;=\;{\mathrm{AUC}}_{\mathrm{Sample}}/{\mathrm{AUC}}_{\mathrm{white}\;\mathrm{bread}}\;\times \;100\%$$

(7) eGI=8.198+0.862 × HI

where AUC_sample_ and AUC_white bread_ represented the areas under the hydrolysis curves of the sample and the reference (fresh white bread), respectively. An eGI value below 55 was classified as low glycemic index, between 55 and 70 as medium, and above 70 as high.

The starch digestion curves were fitted to the parallel first-order kinetic model developed previously by Li et al. [25] according to the equation as follows:

(8) $$C_{t}\;=\;C_{0}\;+\;(C_{1\infty })\;\times \;(1\;-\;e^{-k_{1}t})\;+\;(C_{2\infty })\;\times \;(1\;-\;e^{-k_{2}t})$$

where C_t_ (%) represents the portion of total starch that had been digested at a specific time t (min). C_1∞_ (%) and C_2∞_ (%) denote the estimated percentages of total starch digested by the conclusion of the reaction period for parallel fractions 1 and 2, respectively. C_0_ signifies the amount of starch digested at t = 0. The digestion rate constants for each starch fraction were denoted as k_1_ (min^−1^) and k_2_ (min^−1^). The values for k_1_, k_2_, C_1∞_, and C_2∞_ were determined using a non-linear least square refinement process in Excel to reach a global minimum, while C_0_ was obtained from direct experimental measurements of starch digestion at time 0.

### 2.7. Statistical Analysis

All determinations were carried out in triplicate using independent biological replicates, and results were expressed as mean ± standard deviation. Statistical processing was performed with SPSS software (Version 26, IBM Corp., Armonk, NY, USA). Mean values across independent experimental groups were compared by one-way analysis of variance. Where the ANOVA indicated significant differences, Duncan’s multiple-range test was applied for post hoc pairwise comparisons. We considered a two-tailed *p*-value of less than 0.05 statistically significant. Visual representations were created using Origin software (release 2026, OriginLab Corporation, Northampton, MA, USA).

## 3. Results and Discussion

### 3.1. Amylose Leaching Rate and Swelling Rate

The effect of HMT on amylose leaching was assessed by the leaching of amylose rate and swelling rate of all samples. The results are shown in Table 1. The leaching rate decreased from 20.9% (control) to 19.9% (HMT-1), 17.5% (HMT-2), and 14.8% (HMT-3). Swelling rate also showed the same decreasing tendency: 16.7% for control, 14.1% for HMT-1, 12.5% for HMT-2 and only 8.2% for HMT-3. This was mainly due to the crystalline rearrangement and granule densification caused by HMT [26]. The mild HMT mainly acted on the amorphous part and moderately disrupted the hydrogen-bond network. Consequently, as gelatinization proceeds, water molecules can permeate the starch structure, enabling the comparatively swift release of amylose molecules. The leaching rate achieved under these conditions is only somewhat reduced compared to the baseline experimental group. With increasingly aggressive processing, the starch chains begin to break down, and there is a notable escalation in the percentage of lower molecular weight amylopectin fragments. During drying, these shorter amylopectin chains reassemble into highly ordered crystalline regions, while the granule’s overall framework solidifies into a denser matrix. Both modifications serve to restrict water penetration and subsequently hinder amylose exudation, resulting in a marked decrease in the leaching rate [27]. In contrast to the treated samples, the unprocessed control exhibits its inherent granular architecture, with amylose yield and swelling characteristics falling midpoint between those of mildly and profoundly treated starches. This highlights a clear divergence in starch response to varying processing intensities.

### 3.2. Effects of HMT on the Multi-Scale Structure of Starch

#### 3.2.1. Particle Size

Particle size values are presented in Table 2. The sample of HMT-0 yielded a mean particle size of 6.04 μm, which is consistent with the particle size of native starch granules. Once more we observed an apparent trend for the particle size to increase with the increase in HMT intensity. HMT-1 yielded 7.44 μm, about 23.2% higher than the control. HMT-3 yielded 10.33 μm, around a 71.0% increase compared with the untreated sample, and this was also the largest size in all groups. The increase in particle size was attributed to the following concurrent phenomena. Throughout the elaborate HMT procedure, the granules experienced partial gelatinization. Amorphous sectors within these particles avidly absorbed moisture, leading to their expansion. With escalating treatment strength, the gelatinization process became more extensive, prompting the granules to clump together and form attachments. Concurrently, amylose was released from the granules and redeposited onto their external surfaces. These combined effects resulted in the observed 1.5–9% expansion of the particle size [28].

#### 3.2.2. Long-Range Ordered Structure

**Figure 1:**
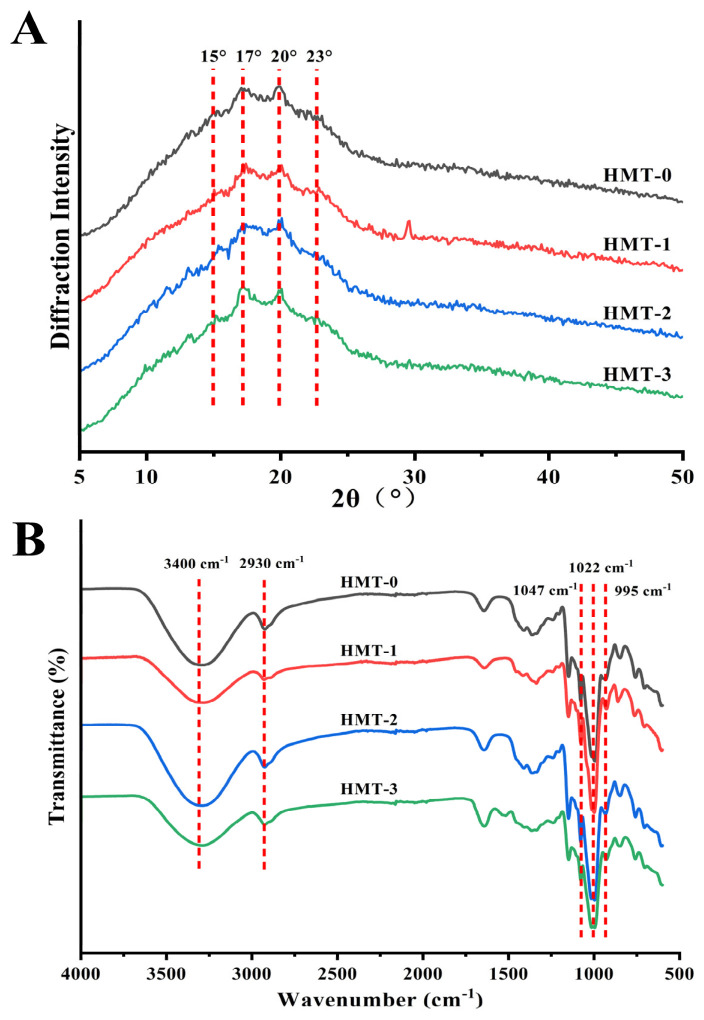
The effects of different HMTs on the long-range order (**A**) and short-range order (**B**) of starch.

XRD patterns are shown in Figure 1. We could find that, in the control sample, there were well-defined characteristic diffraction peaks of A-type starch centered at 2*θ* = 13.10°, 15.46°, 19.95°, 23.49°. The HMT-3 sample displayed a new peak near 2*θ* ≈ 20°, which was suggestive of V-type crystal forms typically associated with amylose–lipid complexes. In comparison with the control, the relative crystallinity of all HMT-treated samples decreased (Table 1). Compared to the control, the direct reason for the decrease was the break of the original hydrogen-bond network inside the starch granules with the synergistic action of moisture and the thermal energy during HMT. The observed process partially unwound the complex double-helix configuration and disrupted the regular molecular pattern [29]. Coinciding with this, the HMT-3 sample showed a notable shift from an A-form to a V-form. This phase change suggests that, as the polymer chains broke down, the released amylose fragments had a strong tendency to bind with naturally occurring lipids within the system. Consequently, they organized into stable single-stranded complexes, specifically inclusion structures, which possessed increased resistance to elevated temperatures. The development of these inclusion complexes marks a pivotal stage within the broader reaction [30]. In summary, it was evident that the extent to which the crystalline architecture was disordered showed a consistent positive correlation with the level of applied treatment, signifying a direct proportionality between these two variables. The unambiguous appearance of the V-type structure was considered as an indisputable sign of molecular reorganization under the intense treatment.

#### 3.2.3. Short-Range Ordered Structure

The infrared spectroscopic analysis is presented in Figure 1 and Table 1. Results showed that HMT did not alter the chemical nature of the starch because the absorption peak shapes were consistent across all samples. However, the short-range order structure parameters R_1047/1022_ and R_1022/995_ changed regularly with increasing treatment intensity. The value of R_1047/1022_ in the control was 0.887. After treatment with HMT, its value decreased to 0.870–0.884 and for the HMT-3 group to 0.870. The trend for R_1022/995_ was also present. The R_1022/995_ value generally increased with HMT intensity, reaching 0.945 in the HMT-3 group. This increasing tendency showed that the HMT stimulated the rearrangement of the starch molecular chains at the interface between amorphous and crystalline regions, leading to more compact double-helix stacking [31]. The decreased in R_1047/1022_ from 0.887 to 0.870 indicated a reduction in short-range order, consistent with the decreased relative crystallinity observed by XRD. The increase in R_1022/995_ from 0.912 to 0.945 reflected an increased proportion of amorphous-related vibrations, which was also consistent with structural disorganization after HMT. The increased proportion of short-chain branched starch provided the molecular basis for this increase in short-range order. Short-chain molecules had less steric hindrance and could more easily form stable double-helix structures through hydrogen bonds, as reported [32].

#### 3.2.4. Amylopectin Chain Length Distribution

The distribution of amylopectin chain lengths results is summarized in Table 2. In the control group, short chains (DP 6–12) made up 28.7%. Long chains (DP 25–36) accounted for 12.6% and ultra-long chains (DP > 36) accounted for 5.3%. After HMT, the proportion of short chains increased and that of long chains correspondingly decreased. Specifically, the proportion of short chains (DP 6–12) rose to a maximum of 39.4%, followed by a drop in the share of long chains (DP 25–36) to a minimum of 8.5%, and the proportion of chains with DP larger than 36 decreased to a range of only 2.8–3.9%. After HMT, the proportion of short chains (DP 6–12) increased from 28.7% to 39.4%, while long chains (DP > 36) decreased from 5.3% to 2.8% (Table 2). This suggested that HMT caused the cleavage of amylopectin long chains and thus more short chains are made. Short-chain molecules have lower steric hindrance and readily form dense double-helix stacks via hydrogen bonds during drying, which contributed to the changes in short-range order observed after HMT [33]. When short chains were present in large excess, they disrupted the regular arrangement of the long-range lattice. As a result, the relative crystallinity decreased, which was in agreement with the decrease in crystallinity noted in the XRD patterns. Moreover, the more short chains there were, the more they inhibited starch retrogradation. The reason was that short chains could not form a stable three-dimensional network structure, and this retarded the process of aging [34]. The samples with chain lengths similar to those of native starch exhibited gelatinization behavior and texture properties closer to those of untreated rice. In contrast, samples with a significantly greater proportion of short chains have lower FV and higher RS [35].

#### 3.2.5. Molecular Weight

The values of M_w_ obtained are given in Table 2. The control group exhibited M_w_ of 1.7 × 10^7^ g/mol, a value consistent with the high molecular weight expected for native starch. After HMT, M_w_ decreased in all groups and the degree of decrease became more remarkable with rising treatment intensity. HMT-1 showed an M_w_ of 1.5 × 10^7^ g/mol, an 11.8% decrease from the control, which indicated that the chains remained largely intact. HMT-2 yielded a value of 1.2 × 10^7^ g/mol, a decrease of 29.4%. HMT-3 presented an M_w_ of 0.9 × 10^7^ g/mol, which represented a 47.1% drop relative to the untreated sample. These results confirmed that HMT breaks starch molecular chains, and to what extent the breakage completed chain scission also increased with increasing severity of treatment.

The decrease in M_w_ was closely correlated to the change in amylopectin chain length distribution. The fraction of short chains (DP 6–12) increased from 28.7% in the control to 39.4% in HMT-3, while long chains (DP ≥ 25) showed a corresponding decrease. The higher number of short chains also directly indicated that molecular chain breakage occurred. If long chains were cut into short ones, the molecular weight inevitably fell. The M_w_ decrease did not follow a simple linear pattern, indicating the existence of regions of the starch chains that were relatively resistant to hydrolysis [36]. Lower Mw also affected gelatinization behavior: as chains shortened, the ability of amylose to reassociate on cooling was diminished and the tendency to retrogradation was thereby suppressed [37].

### 3.3. Effect of HMT on the Functional Properties of Starch

#### 3.3.1. Viscosity Property

The viscosity properties of starches treated with different HMTs are shown in Table 3. The control starch had a peak viscosity of 2488 cP, a breakdown value of 192 cP, and a pasting temperature of 84.18 °C. After HMT, the viscosity parameters generally decreased, while the gelatinization temperatures generally increased. The pasting temperature of the HMT1 sample was 87.43 °C, similar to that of the control group. Its peak viscosity was 1924 cP, and the breakdown value was 199 cP. As the HMT intensity increased, the peak viscosity decreased to between 1470 cP and 1535 cP, and the gelatinization temperature rose to between 87.70 °C and 90.95 °C. HMT caused rearrangement and recrystallization of starch molecular chains, which enhanced the structural compactness and crystalline stability of starch granules. This inhibited excessive granule swelling during gelatinization, thus reducing the peak viscosity and breakdown value. Meanwhile, it delayed the reassociation of amylose and effectively suppressed starch retrogradation [38]. Furthermore, the complexes formed between amylose and lipids restricted the reassociation of starch chains during cooling, reducing the setback viscosity from 1362 cP to between 970 cP and 1359 cP. The increase in gelatinization temperature reflected better thermal stability of the crystalline regions, requiring more energy to disrupt the reorganized crystalline structures [39].

#### 3.3.2. Thermodynamic Properties

The thermodynamic properties of the HMT-treated starches are summarized in Table 3. For the HMT-0 group, T_o_, T_p_, and ΔH were 42.71 °C, 49.62 °C, and 2.11 J/g, respectively. After HMT, the thermodynamic parameters changed in a way that depended on the treatment intensity. For the HMT-1 group, T_o_ increased slightly to 45.32 °C, while ΔH dropped markedly to 1.29 J/g. In contrast, the HMT-2 and HMT-3 groups showed much higher T_o_ (60.64–61.44 °C), T_p_ (67.81–68.08 °C), T_c_ (73.55–74.31 °C) and ΔH (5.33–5.92 J/g) than the control. The increases in T_o_, T_p_ and T_c_ indicated better thermal stability of the crystalline regions after intensive HMT, meaning more energy was required to disrupt the reorganized crystalline structures [40]. The rise in ΔH suggested that HMT promoted the formation of more ordered double-helical structures through rearrangement of short chains, which agreed with the higher R_1022/995_ values seen in the FT-IR analysis [41]. The higher T_o_ and ΔH values in HMT-2 and HMT-3 thus illustrated the combined effect of crystalline phase transformation and enhanced molecular order induced by HMT [42]. The biphasic change in ΔH deserves further explanation. Under mild HMT (HMT-1, 100 °C), the applied heat and moisture partially disrupted the native hydrogen-bond network and double-helical order, leading to a decrease in ΔH from 2.11 J/g (control) to 1.29 J/g. In contrast, under stronger HMT (HMT-2 and HMT-3, 110–120 °C), the increased proportion of short amylopectin chains (DP 6–12 increased to 36.3–39.4%) and the formation of V-type starch–lipid complexes promoted recrystallization into more ordered and thermostable structures, which significantly increased ΔH to 5.33–5.92 J/g. As already discussed in Section 3.2.4, the increased proportion of short amylopectin chains under stronger HMT promoted recrystallization, which directly explained the increased ΔH observed here. This interpretation was consistent with the increased R_1022/995_ ratio (0.919–0.945) and the appearance of V-type peaks in XRD patterns.

#### 3.3.3. Rheological Properties

**Figure 2:**
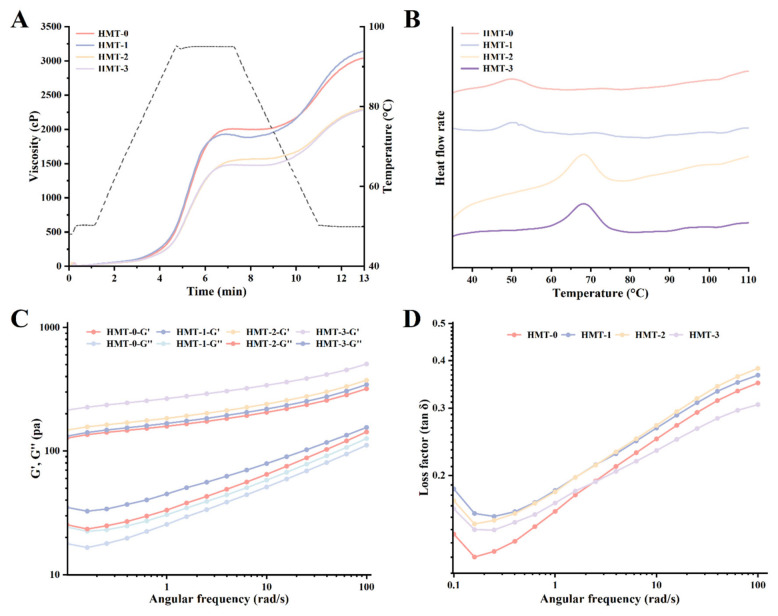
Functional properties of starch from HMT-treated rice. (**A**) Pasting curves from rapid visco analysis (RVA); (**B**) DSC thermograms; (**C**) Frequency sweep of storage modulus (G′) and loss modulus (G″) at 25 °C; (**D**) Loss tangent (tan δ = G″/G′) as a function of frequency.

The results of the paste rheological properties for different HMT-treated starches are shown in Figure 2C,D. The G′ of all samples was higher than G″, indicating predominantly elastic (solid-like) gel behavior. The control group had a G′ of 482.50 Pa, while G′ decreased overall after HMT. Specifically, the HMT-1 group had a G′ of 538.20 Pa, which was actually higher than that of the control group, while its tanδ was 0.16, the lowest among all groups. This indicated that this treatment preserved a strong hydrogen-bond network and likely enhanced starch–protein interactions. G′ values in the other HMT groups varied from 210.50 to 410.30 Pa. The HMT-3 group had a G′ value of 385.60 Pa and a tanδ of 0.18, indicating good gel network stability even under higher treatment intensities. Changes in rheological behavior are highly correlated with the extent of cross-linking of the starch molecular chains and the crystalline structure [43]. In the HMT-1 group, moderate treatment enhanced complexation of amylose with lipids. CLSM images showed increased yellow fluorescence (indicating starch–protein co-localization), which is qualitatively consistent with possible protein denaturation and additional interactions; this may partially explain why G′ increased rather than decreased. Conversely, high-intensity HMT significantly reduced the M_w_ of starch, increased the proportion of short chains, and reduced interchain entanglement, resulting in a lower overall G′ [44]. However, in the HMT-3 group, the rearrangement of short-chain molecules formed dense crystalline regions, partially compensating for the network weakening caused by the shortened chain length; consequently, tanδ remained at a low level.

### 3.4. Effects of HMT on Starch Digestibility

**Figure 3:**
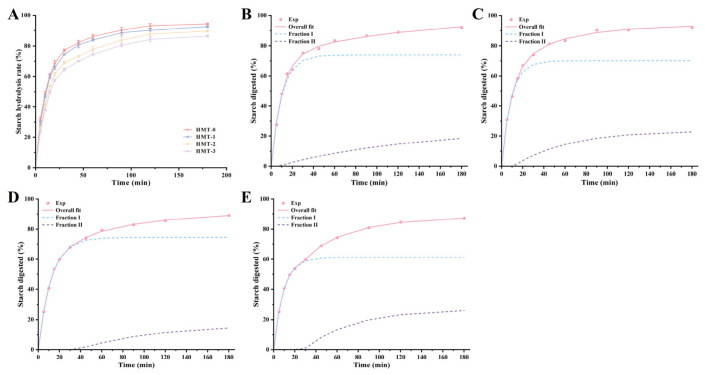
In vitro digestion and kinetic fitting diagram of HMT rice starch. (**A**) Digestion curve of native starch; (**B**–**E**) First-order digestion kinetics fitting curves for HMT-0, HMT-1, HMT-2, and HMT-3, respectively.

In vitro digestibility data are presented in Figure 3 and Table 4. The control sample contained 68.7% RDS and 6.9% RS. After HMT, RDS declined while RS increased. In the HMT-3 group RDS was 57.4% and RS was 15.8%, representing more than a doubling of the resistant fraction. These changes were closely linked to the multi-scale structural reorganization of starch brought about by HMT. The treatment caused partial degradation of starch chains, raising the proportion of short-chain amylopectin. During drying they (the short chains) rearranged into the more compact crystalline regions [7]. At the same time, heat-denatured proteins may form a composite matrix with starch, potentially creating a physical barrier on the granule surfaces and in the particles as previously suggested [8,45]. The qualitative CLSM observations (yellow fluorescence indicating starch–protein co-localization), together with the observed changes in pasting and rheological properties, were consistent with the possibility that HMT promoted protein denaturation and starch–protein interactions [8]. In native starch, enzymatic hydrolysis relies on enzyme adsorption to the granule surface followed by inward diffusion. The surface coverage after HMT became more compact and the particle size was increased, both contributing to an increase in resistance to enzyme–substrate contact considerably. As a result, RS content showed a substantial increase [46].

After the samples were thoroughly gelatinized, the RDS of all samples increased greatly, while the RS decreased notably. In the gelatinized control sample, the RDS was 68.7% and the RS was 6.9%. In contrast, the RS in the HMT-treated group remained at a relatively high level after gelatinization, ranging from 6.9% to 15.8%, which was about 4.1 times that of the control. The gelatinization process disrupted the long-range ordered structure of starch granules and caused most crystalline regions to melt. This residual resistance was attributed to thermally stable V-type amylose–lipid complexes that do not completely dissociate at gelatinization temperatures [47]. Additionally, during the cooling phase after gelatinization, short branched amylose chains reassociate into ordered structures, forming retrograde resistant starch (RS3) [48]. Furthermore, the HMT-induced starch–protein cross-linking network remained partially intact after heating and continued to hinder the binding of digestive enzymes to the substrate [49]. These results suggested that HMT improved starch resistance to enzymatic hydrolysis by acting synergistically at various scales; such as modification of molecular chain length distribution, crystalline rearrangement and matrix densification. This resistance was partially preserved even after gelatinization.

## 4. Conclusions

This investigation has explored the effect of HMT in regulating the multi-scale structure, functional properties, and digestibility of low-GI rice starch. Treatment at 120 °C degraded starch molecular chains, leading to a lower Mw and a broader molecular weight distribution. The fraction of short amylopectin chains increased and that of long chains decreased rapidly upon reduction. Relative crystallinity declined, but short-range molecular order improved, and the particle size increased. These structural changes led to reduced peak viscosity, higher gelatinization temperature and enthalpy, and altered gel strength (increased after mild HMT but decreased after intense HMT). As a result of the mixed effect of all these structural and functional changes, starch digestibility was modified: the treated starch had less RDS, more SDS and RS, a low eGI, and a considerably longer lag time before slow digestion was reached. To establish a clearer mechanistic link, the observed structural changes directly explain the digestibility results. An increased proportion of short-chain amylopectin (DP 6–12) and a decrease in molecular weight (M_w_) led to a reduction in gelatinization viscosity and an enhancement of short-range order. The formation of V-type starch–lipid complexes and an increased R_1022/995_ ratio contributed to improved thermal stability and enzyme resistance. These results scientifically support the use of HMT to produce low-GI products from rice starch.

## Figures and Tables

**Table 1:** Effects of different HMT on the amylose leaching rate, swelling rate, crystallinity, short-range order, and full width at half maximum of rice starch.

Sample	Leaching Rate (%)	Swelling Rate (%)	Crystallinity (%)	R_1047/1022_	R_1022/995_	FWHM-480
HMT-0	20.9 ± 0.6 ^a^	16.7 ± 0.1 ^a^	24.3 ± 0.5 ^a^	0.887 ± 0.004 ^a^	0.912 ± 0.006 ^b^	11.99 ± 0.01 ^b^
HMT-1	19.9 ± 0.1 ^a^	14.1 ± 0.2 ^b^	18.9 ± 0.7 ^b^	0.884 ± 0.012 ^a^	0.916 ± 0.017 ^b^	12.09 ± 0.00 ^b^
HMT-2	17.5 ± 0.1 ^b^	12.5 ± 0.2 ^c^	17.7 ± 0.7 ^c^	0.873 ± 0.011 ^b^	0.919 ± 0.009 ^b^	12.19 ± 0.00 ^b^
HMT-3	14.8 ± 0.2 ^c^	8.2 ± 0.1 ^d^	15.8 ± 0.8 ^d^	0.870 ± 0.006 ^b^	0.945 ± 0.021 ^a^	15.07 ± 0.04 ^a^

Note: R_1047_/_1022_, ratio of absorbance at 1047 cm^−1^ to 1022 cm^−1^; R_1022_/_995_, ratio of absorbance at 1022 cm^−1^ to 995 cm^−1^; FWHM-480, full width at half maximum of the Raman band at 480 cm^−1^. Data are expressed as mean ± SD (*n* = 3). Different superscript letters in the same column indicate significant differences (*p* < 0.05).

**Table 2:** Effects of different HMTs on the particle size, molecular weight, and amylopectin chain length distribution of rice starch.

Sample	Particle Size (μm)	M_w_ (×10^7^ g/mol)	PDI	DP 6–12 (%)	DP 13–24 (%)	DP 25–36 (%)	DP > 36 (%)
HMT-0	6.04 ± 0.02 ^d^	1.72 ± 0.03 ^a^	1.86 ± 0.01 ^d^	28.7 ± 0.2 ^d^	53.4 ± 0.3 ^a^	12.6 ± 0.2 ^a^	5.3 ± 0.1 ^a^
HMT-1	6.11 ± 0.03 ^c^	1.46 ± 0.07 ^b^	2.65 ± 0.03 ^c^	32.6 ± 0.2 ^c^	52.3 ± 0.2 ^b^	11.2 ± 0.1 ^b^	3.9 ± 0.1 ^b^
HMT-2	6.32 ± 0.03 ^b^	1.23 ± 0.09 ^c^	3.7 ± 0.29 ^b^	36.3 ± 0.2 ^b^	50.5 ± 0.3 ^c^	9.6 ± 0.3 ^c^	3.6 ± 0.2 ^b^
HMT-3	6.51 ± 0.03 ^a^	0.92 ± 0.02 ^d^	5.14 ± 0.12 ^a^	39.4 ± 0.2 ^a^	49.4 ± 0.3 ^d^	8.5 ± 0.2 ^d^	2.8 ± 0.3 ^c^

Note: M_w_, weight-average molecular weight; PDI, polydispersity index; DP 6–12, degree of polymerization 6–12; DP 13–24, degree of polymerization 13–24; DP 25–36, degree of polymerization 25–36; DP > 36, degree of polymerization > 36. Data are expressed as mean ± SD (*n* = 3). Different superscript letters in the same column indicate significant differences (*p* < 0.05).

**Table 3:** Effects of different HMTs on the viscosity and thermodynamic properties of starch.

Sample	PV (cP)	TV (cP)	FV (cP)	SV (cP)	BV (cP)	GT (°C)	T_o_ (°C)	T_p_ (°C)	T_c_ (°C)	ΔH (J/g)
HMT-0	2488 ± 684 ^a^	2296 ± 703 ^a^	3658 ± 857 ^a^	1362 ± 155 ^a^	192 ± 29 ^b^	84.18 ± 4.81 ^c^	42.71 ± 0.07 ^b^	49.77 ± 0.27 ^c^	56.26 ± 0.18 ^b^	2.11 ± 0.08 ^c^
HMT-1	1924 ± 67 ^b^	1726 ± 79 ^b^	3085 ± 112 ^b^	1359 ± 43 ^a^	199 ± 44 ^b^	87.43 ± 0.37 ^b^	45.32 ± 0.20 ^b^	50.30 ± 0.03 ^b^	55.74 ± 0.14 ^b^	1.29 ± 0.10 ^d^
HMT-2	1535 ± 5 ^c^	1315 ± 22 ^c^	2343 ± 31 ^c^	1028 ± 8 ^b^	220 ± 20 ^a^	90.38 ± 0.02 ^a^	60.64 ± 0.08 ^a^	67.92 ± 0.11 ^a^	73.55 ± 0.07 ^a^	5.92 ± 0.14 ^a^
HMT-3	1470 ± 19 ^d^	1277 ± 19 ^d^	2247 ± 32 ^c^	970 ± 14 ^c^	193 ± 8 ^b^	90.95 ± 0.35 ^a^	61.44 ± 0.09 ^a^	68.02 ± 0.05 ^a^	74.31 ± 0.02 ^a^	5.33 ± 0.06 ^b^

Note: PV, peak viscosity; TV, trough viscosity; FV, final viscosity; SV, setback viscosity; BV, breakdown viscosity; GT, gelatinization temperature; T_o_, onset temperature of gelatinization; T_p_, peak temperature of gelatinization; T_c_, conclusion temperature of gelatinization; ΔH, gelatinization enthalpy. Data are expressed as mean ± SD (*n* = 3). Different superscript letters in the same column indicate significant differences (*p* < 0.05).

**Table 4:** In vitro digestion and kinetic fitting parameters of HMT rice starch.

Sample	RDS (%)	SDS (%)	RS (%)	HI (%)	eGI	k_1_ (min^−1^)	k_2_ (min^−1^)	C_1∞_ (%)	C_2∞_ (%)	T_2start_ (min)
HMT-0	68.7 ± 2.0 ^a^	24.5 ± 0.5 ^b^	6.9 ± 1.5 ^c^	53.4 ± 0.5 ^a^	54.19 ± 0.43 ^a^	0.11 ± 0.01 ^a^	0.02 ± 0.00 ^a^	75.5 ± 0.5 ^a^	20.4 ± 0.4 ^b^	14.15 ± 0.25 ^b^
HMT-1	65.8 ± 1.2 ^b^	24.4 ± 0.8 ^b^	9.8 ± 1.0 ^b^	52.0 ± 0.5 ^b^	53.00 ± 0.40 ^b^	0.10 ± 0.01 ^a^	0.01 ± 0.00 ^b^	72.1 ± 1.9 ^b^	21.8 ± 3.0 ^b^	16.57 ± 5.13 ^b^
HMT-2	61.4 ± 1.2 ^c^	26.3 ± 0.3 ^ab^	12.3 ± 1.5 ^a^	49.3 ± 0.5 ^c^	50.66 ± 0.39 ^c^	0.09 ± 0.00 ^a^	0.01 ± 0.00 ^b^	70.6 ± 1.8 ^b^	23.6 ± 1.8 ^b^	20.96 ± 1.42 ^b^
HMT-3	57.4 ± 0.9 ^d^	26.8 ± 0.7 ^a^	15.8 ± 1.5 ^c^	47.1 ± 0.4 ^d^	48.80 ± 0.38 ^d^	0.11 ± 0.00 ^a^	0.02 ± 0.00 ^a^	61.6 ± 0.9 ^c^	26.9 ± 1.0 ^a^	28.62 ± 1.40 ^a^

Note: RDS signifies rapidly digestible starch, a kind that is processed quickly during digestion. In contrast, SDS, which stands for slowly digestible starch, is metabolized at a slower rate. RS denotes resistant starch, a form that largely avoids being broken down. HI refers to the hydrolysis index, while eGI denotes the estimated glycemic index. The values k_1_ and k_2_ indicate the rate constants for the initial and secondary digestion phases, respectively. C_1∞_ and C_2∞_ signify the maximal starch digestion levels for each phase as time approaches infinity. T_2start_ measures the delay before the slower fraction begins to digest. Results are reported as the average ± standard deviation (*n* = 3). Within each category, rows marked with distinct superscripts indicate statistically significant disparities (*p* < 0.05).

## Data Availability

The original contributions presented in this study are included in the article. Further inquiries can be directed to the corresponding author.
